# The Potency of Cytotoxic Mechanisms of Local Anesthetics in Human Chondrocyte Cells

**DOI:** 10.3390/ijms252413474

**Published:** 2024-12-16

**Authors:** Jia-Lin Chen, Shu-Ting Liu, Chia-Chun Wu, Yi-Chou Chen, Shih-Ming Huang

**Affiliations:** 1 Department of Anesthesiology, Tri-Service General Hospital, National Defense Medical Center, Taipei 114, Taiwan; babe.ane@gmail.com; 2 Department of Biochemistry, National Defense Medical Center, Taipei 114, Taiwan; shuting0719@gmail.com; 3 Department of Orthopedics, Tri-Service General Hospital, National Defense Medical Center, Taipei 114, Taiwan; doc20281@mail.ndmctsgh.edu.tw; 4 Department of Orthopedics, Taoyuan General Hospital, Ministry of Health and Welfare, Taoyuan 330, Taiwan

**Keywords:** lidocaine, levobupivacaine, bupivacaine, ropivacaine, chondrotoxicity, apoptosis, proliferation, autophagy, active pharmaceutical ingredients

## Abstract

Local anesthetics are commonly used in various clinical settings for both prevention and symptom relief. Numerous clinical studies have demonstrated that intra-articular injections of local anesthetics achieve high success rates in orthopedic practices. However, several widely used local anesthetics, including bupivacaine, lidocaine, and ropivacaine, have been shown to exhibit toxicity to chondrocytes, with the underlying mechanisms of chondrotoxicity remaining poorly understood. In this study, we aimed to investigate the cytotoxic effects of local anesthetics, specifically focusing on the consequences of a single intra-articular injection in human chondrocyte cells. Our results reveal that lidocaine, levobupivacaine, bupivacaine, and ropivacaine induced cell death, characterized by the induction of apoptosis and the suppression of cellular proliferation. These effects were mediated through mechanisms involving oxidative stress, mitochondrial dysfunction, and autophagy pathways. We found that the toxic effects of local anesthetics were concentration-dependent, with lidocaine exhibiting the lowest cytotoxicity among the tested agents in TC28a cells. Notably, bupivacaine and levobupivacaine displayed significant cytotoxic effects related to apoptosis, cellular proliferation, reactive oxygen species generation, mitochondrial membrane potential depolarization, and autophagy in human chondrocyte cells. Our findings not only support existing clinical studies but also highlight potential targets for developing protective agents to mitigate serious side effects associated with their use in orthopedic practices.

## 1. Introduction

Local anesthetics function by binding to and inhibiting voltage-gated sodium channels in nerve cell membranes, which prevents the formation of action potentials and blocks nerve transmission [1,2,3,4]. Local anesthetics are routinely utilized in various clinical scenarios for both prevention and symptomatic relief [5]. The most serious side effect associated with local anesthetics is cardiac and central nervous system (CNS) toxicity [6]. Managing acute postoperative pain following orthopedic surgery poses challenges for anesthetists and surgeons alike. The delivery of local anesthetics into the joint space, whether via a single injection or continuous infusion, has emerged as an established technique for postoperative analgesia, especially after arthroscopic procedures [7,8]. In orthopedic practice, these agents can be administered through various methods—including spinal, epidural, or intra-articular routes—to alleviate postoperative pain or to treat osteoarthritis. Numerous clinical studies have demonstrated that intra-articular injections of local anesthetics yield high success rates for postoperative pain management [7,8]. In clinical settings, local anesthetic toxicity is most commonly observed with continuous infusions via pain pumps in human chondrocytes and tenocytes [9,10]. Yet, laboratory findings suggest that even a single exposure to local anesthetics may pose risks to cartilage health [11,12].

Local anesthetics are primarily categorized into two types: esters and amides. Common examples of amides include lidocaine, bupivacaine, and ropivacaine, which are frequently used in peripheral joint injections [2,8,13]. Local anesthetics can be differentiated based on various chemical characteristics such as lipid solubility, protein binding, and the acid dissociation constant, all of which significantly influence their potency, duration of action, and onset time [2,8]. Notably, inflamed tissue tends to be more acidic than healthy tissue, which can diminish both protein binding and the availability of the nonionized drug, which is crucial for achieving effective analgesia. The potency of the three local anesthetics is closely linked to their lipid solubility, which is lowest in lidocaine and highest in bupivacaine [2,8]. Lidocaine boasts the lowest acid dissociation constant (pKa) and protein solubility, resulting in the fastest onset of action (less than 2 min) and the shortest duration of action (30 to 120 min) [14]. By contrast, bupivacaine exhibits the slowest onset (2 to 10 min) and the longest duration (180 to 360 min) [15]. Ropivacaine matches bupivacaine’s onset time due to similar pKa values, while its duration of action typically falls between those of lidocaine and bupivacaine (140 to 200 min) [16]. Although existing studies have assessed the impact of local anesthetics on human cartilages over designated timeframes ranging from 15 min to 24 h, and many have compared multiple agents, these studies were conducted in vitro, limiting our ability to draw conclusive insights regarding the in vivo effects of a single intra-articular injection. Bupivacaine is particularly popular for intra-articular analgesia due to its extended duration of action [17]. Despite its prevalent use, the effects of intra-articular local anesthetic agents on joint structures remain incompletely understood.

Local anesthetics can be administered as a single intra-articular injection during or after a procedure, or through postoperative pain pumps for extended relief. Recent studies indicate that local anesthetics can be toxic to human chondrocytes and tenocytes [8,17]. Chondrolysis is a condition characterized by a significant loss of articular cartilage within a relatively brief period [18,19,20]. The level of local anesthetic toxicity is contingent upon factors such as the drug type, concentration, use of adjunctive medications (including steroids or epinephrine), and underlying tissue conditions [6,21]. Chondrocyte dysfunction and death are early indicators of osteoarthritis [22,23]; thus, evidence of local anesthetic chondrotoxicity necessitates further investigation into the potential long-term clinical implications of intra-articular local anesthetic exposure. While laboratory studies have shown that prolonged contact with concentrated local anesthetics is harmful to cartilages, the clinical relevance—particularly regarding a single intra-articular injection—remains ambiguous. Recent research has highlighted that several widely used local anesthetics, including bupivacaine, lidocaine, and ropivacaine, exhibit toxicity to both human and animal chondrocytes in vitro and in vivo [24,25]. Nonetheless, the underlying mechanisms of chondrotoxicity remain poorly understood.

In this study, we aimed to investigate the mechanisms underlying the cytotoxic effects of local anesthetics, particularly focusing on the impact of a single intra-articular injection. Four popular local anesthetics, namely lidocaine, levobupivacaine, bupivacaine, and ropivacaine, were examined for their potency and working mechanisms to elucidate chondrocyte dysfunction and death using human chondrocyte cells. Our findings support current clinical studies and provide potential targets for the development of protecting agents to avoid the serious side effects in orthopedic practices.

## 2. Results

### 2.1. The Cytotoxic Pathways of Lidocaine, Levobupivacaine, Bupivacaine, and Ropivacaine Were Investigated in Human TC28a2 Chondrocyte Cells

**Figure 1:**
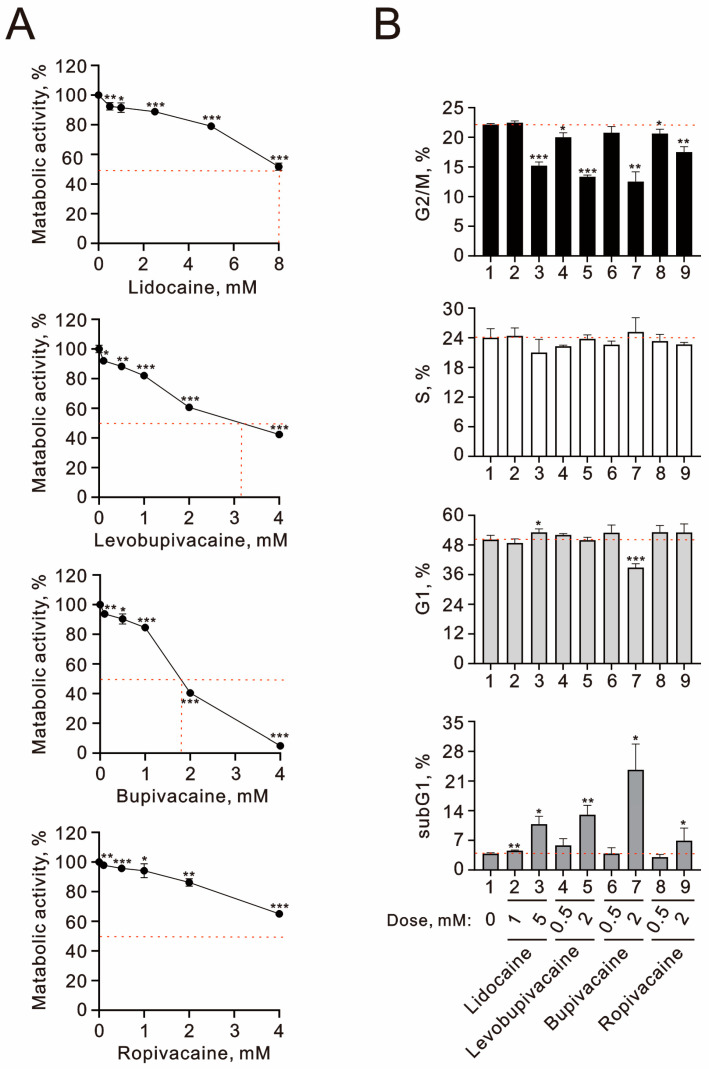
The effects of local anesthetics on the cell viability and cell cycle profile in human TC28a2 chondrocyte cells. (**A**) TC28a2 cells were treated with the specified concentrations of lidocaine (0.5, 1, 2.5, 5, and 8 mM), levobupivacaine (0.1, 0.5, 1, 2, and 4 mM), bupivacaine (0.1, 0.5, 1, 2, and 4 mM), and ropivacaine (0.1, 0.5, 1, 2, and 4 mM) for 24 h. Control cells were maintained under identical conditions. Metabolic activity was assessed using an MTT colorimetric assay, with data expressed as a percentage of the control. The dashed red line indicates the 50% level of the vehicle control. (**B**) TC28a2 cells were treated with the indicated concentrations of lidocaine (1 and 5 mM), levobupivacaine (0.5 and 2 mM), bupivacaine (0.5 and 2 mM), and ropivacaine (0.5 and 2 mM) for 24 h. Following treatment, cells were stained with propidium iodide (PI), a dye that binds to total DNA, allowing for the determination of cell cycle positions based on the intensity of PI staining. The dashed red line represents the level for the vehicle control. The results presented in both (**A**,**B**) are representative of three independent experiments. The bars depict the mean ± SD. * *p* < 0.05; ** *p* < 0.01; *** *p* < 0.001 (Student’s *t*-tests).

In this study, we aimed to explore the mechanisms underlying the cytotoxic effects of local anesthetics, with a particular emphasis on the impact of a single intra-articular injection in human chondrocyte cells. We first assessed the effect of various local anesthetics—including lidocaine, levobupivacaine, bupivacaine, and ropivacaine—on the viability of human TC28a2 chondrocyte cells. A diagram illustrating the local anesthetics lidocaine, levobupivacaine, bupivacaine, and ropivacaine was presented in our previous study [26]. Through a dose–response experiment, we evaluated the detrimental effects of these anesthetics on metabolic activity using the MTT (thiazolyl blue tetrazolium bromide) cell viability assay. Our findings revealed the following order of cytotoxicity among the anesthetics, based on their respective IC_50_ values: lidocaine (IC_50_ ≈ 8 mM), levobupivacaine (IC_50_ ≈ 3.2 mM), bupivacaine (IC_50_ ≈ 2.8 mM), and ropivacaine (IC_50_ ≈ 5 mM) (Figure 1A). Next, we examined how these anesthetics affected the cell cycle profile in TC28a2 cells. Four cell subpopulations (i.e., the subG1, G1, S, and G2/M phases) were distinguished based on DNA content distribution: the cells in the subG1 phase had a lower DNA content (hypodiploid), and the cells in the G1 and G2/M phases were DNA-diploid and -tetraploid, respectively; thus, the G2/M phase fluoresced twice as bright. All tested local anesthetics significantly increased the proportion of cells in the subG1 phase, while markedly reducing the population in the G2/M phase at higher concentrations (Figure 1B). Notably, bupivacaine at a concentration of 2 mM also decreased the G1 phase cell population. The observed cytotoxicity is likely attributed to the induction of apoptosis and suppression of cell proliferation.

**Figure 2:**
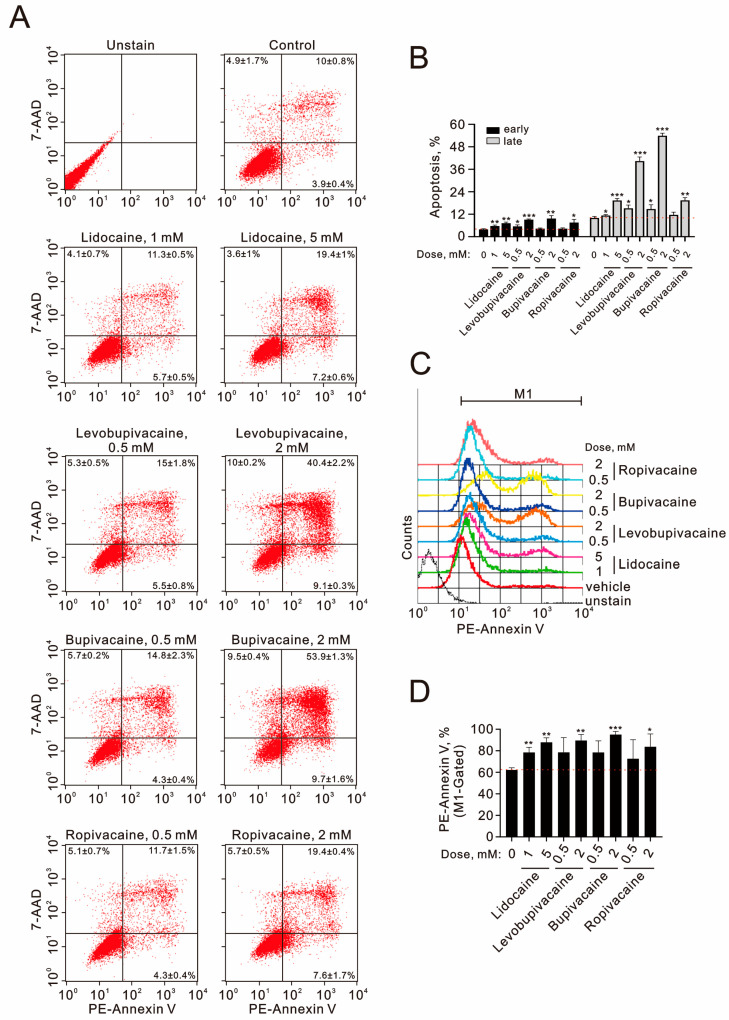
The effects of local anesthetics on early and late apoptosis in human TC28a2 chondrocyte cells. (**A**–**D**) TC28a2 cells were treated with the indicated concentrations of lidocaine (1 and 5 mM), levobupivacaine (0.5 and 2 mM), bupivacaine (0.5 and 2 mM), and ropivacaine (0.5 and 2 mM) for 24 h. Cellular apoptosis was assessed using annexin V apoptosis analysis with 7-AAD staining. In (**A**,**B**), early apoptotic cells are identified as PE–annexin V-positive and 7-AAD negative, while late apoptotic cells are both PE–annexin V-positive and 7-AAD positive. In (**C**,**D**), the intensities of PE–annexin V staining were measured and quantified. The results presented in (**A**,**D**) are representative of three independent experiments. The dashed red line indicates the level for the vehicle control. The symbols and bars depict the mean ± SD. * *p* < 0.05; ** *p* < 0.01; *** *p* < 0.001 (Student’s *t*-tests).

Given the increased subG1 population, we investigated the effects of lidocaine (1 mM and 5 mM), levobupivacaine (0.5 mM and 2 mM), bupivacaine (0.5 mM and 2 mM), and ropivacaine (0.5 mM and 2 mM) on early and late apoptotic stages using the Annexin V assay. Our results indicate that the highest concentrations of lidocaine, levobupivacaine, bupivacaine, and ropivacaine significantly increased both the early and late apoptotic cell populations (Figure 2A,B). Notably, levobupivacaine (2 mM) and bupivacaine (2 mM) produced a greater apoptosis induction compared to lidocaine (5 mM) and ropivacaine (2 mM). The levels of phycoerythrin (PE)–annexin V were significantly elevated by lidocaine (1 mM and 5 mM), levobupivacaine (2 mM), bupivacaine (2 mM), and ropivacaine (2 mM) in TC28a2 cells (Figure 2C,D).

**Figure 3:**
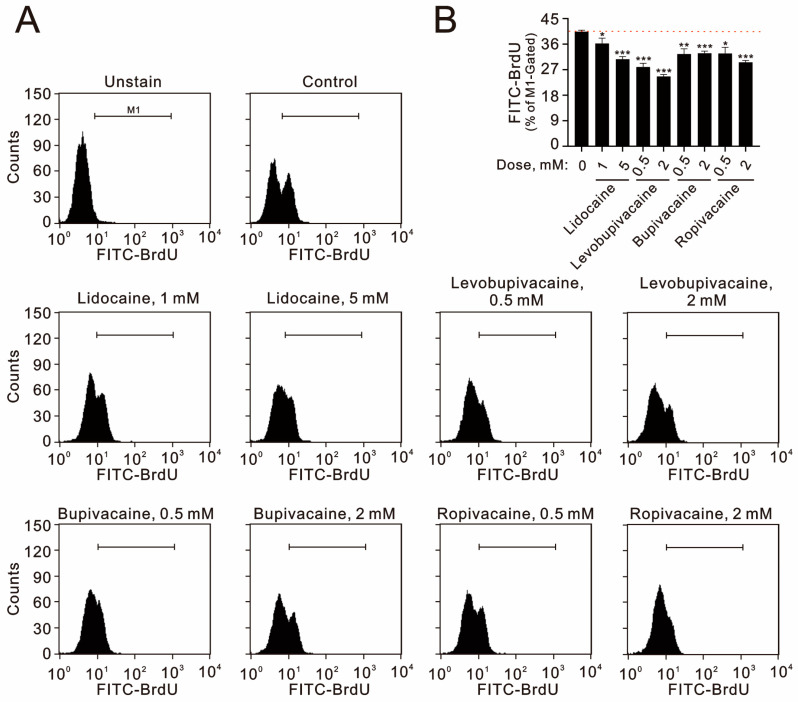
The effects of local anesthetics on cellular proliferation in human TC28a2 chondrocyte cells. (**A**,**B**) TC28a2 cells were treated with the specified concentrations of lidocaine (1 and 5 mM), levobupivacaine (0.5 and 2 mM), bupivacaine (0.5 and 2 mM), and ropivacaine (0.5 and 2 mM) for 24 h. Following treatment, the cells were stained with BrdU, which is incorporated into newly synthesized DNA as the cells progress through the S phase (DNA synthesis). BrdU incorporation levels were then analyzed using flow cytometry. The results presented in (**B**) are representative of three independent experiments. The dashed red line indicates the level for the vehicle control. The bars depict the mean ± SD. * *p* < 0.05; ** *p* < 0.01; *** *p* < 0.001.

Additionally, we assessed the impact of these anesthetics on cellular proliferation using immunofluorescent staining with bromodeoxyuridine (BrdU). Our data demonstrate that all tested concentrations of lidocaine, levobupivacaine, bupivacaine, and ropivacaine significantly inhibited cellular proliferation in TC28a2 cells (Figure 3A,B).

### 2.2. Local Anesthetics Can Induce the Production of Reactive Oxygen Species (ROS), Alter Mitochondrial Membrane Potential, and Stimulate Autophagy in TC28a2 Cells

**Figure 4:**
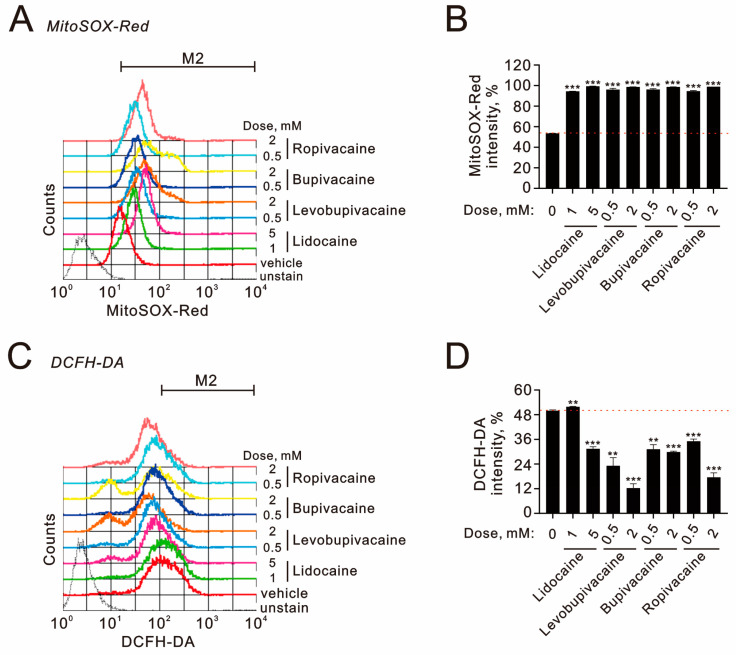
The effects of local anesthetics on mitochondrial and cytosolic ROS in human TC28a2 chondrocyte cells. (**A**–**D**) TC28a2 cells were treated with the specified concentrations of lidocaine (1 and 5 mM), levobupivacaine (0.5 and 2 mM), bupivacaine (0.5 and 2 mM), and ropivacaine (0.5 and 2 mM) for 24 h. Subsequently, they were analyzed for (**A**,**B**) MitoSOX intensity to measure mitochondrial ROS and (**C**,**D**) DCFH-DA intensity to assess cytosolic ROS levels. The results presented in (**B**,**D**) are representative of three independent experiments. The dashed red line indicates the level for the vehicle control. The symbols and bars depict the mean ± SD. ** *p* < 0.01; *** *p* < 0.001 (Student’s *t*-tests).

The cytotoxicity associated with these local anesthetics appears to be linked to ROS generation, changes in mitochondrial membrane potential, and the activation of autophagy. To evaluate these effects, we conducted cytometric analyses using MitoSox for measuring mitochondrial ROS, DCFH-DA for assessing cytosolic ROS, JC-1 dye for evaluating mitochondrial membrane potential, and acridine orange for detecting autophagy in TC28a2 cells. Our assessment of mitochondrial ROS revealed that all tested local anesthetics significantly increased ROS production in mitochondria (Figure 4A,B). With the exception of 1 mM of lidocaine, the other anesthetics significantly reduced cytosolic ROS levels (Figure 4C,D), with levobupivacaine (2 mM) demonstrating the most pronounced inhibitory effect on cytosolic ROS. 

**Figure 5:**
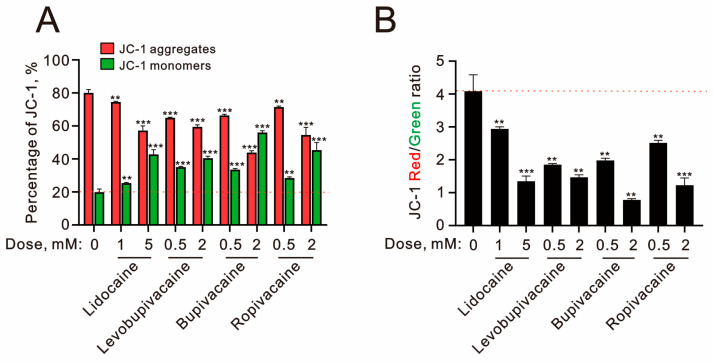
The effects of local anesthetics on mitochondrial membrane potential in human TC28a2 chondrocyte cells. (**A**,**B**) TC28a2 cells were treated with the specified concentrations of lidocaine (1 and 5 mM), levobupivacaine (0.5 and 2 mM), bupivacaine (0.5 and 2 mM), and ropivacaine (0.5 and 2 mM) for 24 h. Mitochondrial membrane potential was evaluated using flow cytometry with JC-1 staining. In (**A**), the percentages of red (aggregates) and green (monomers) fluorescence intensities are presented. In (**B**), the ratios of red to green fluorescence intensities were measured and plotted. The dashed red line indicates the level for the vehicle control. The bars depict the means ± SDs of three independent experiments. ** *p* < 0.01; *** *p* < 0.001 (Student’s *t*-tests).

The analysis of the mitochondrial membrane potential showed that all tested local anesthetics significantly decreased the levels of JC-1 aggregates (red) while increasing the levels of JC-1 monomers (green) (Figure 5A). The observed decrease in the ratio of JC-1 red to JC-1 green supports our conclusion that local anesthetics induce mitochondrial membrane depolarization in TC28a2 cells (Figure 5B), with bupivacaine (2 mM) exhibiting the most substantial effect. 

**Figure 6:**
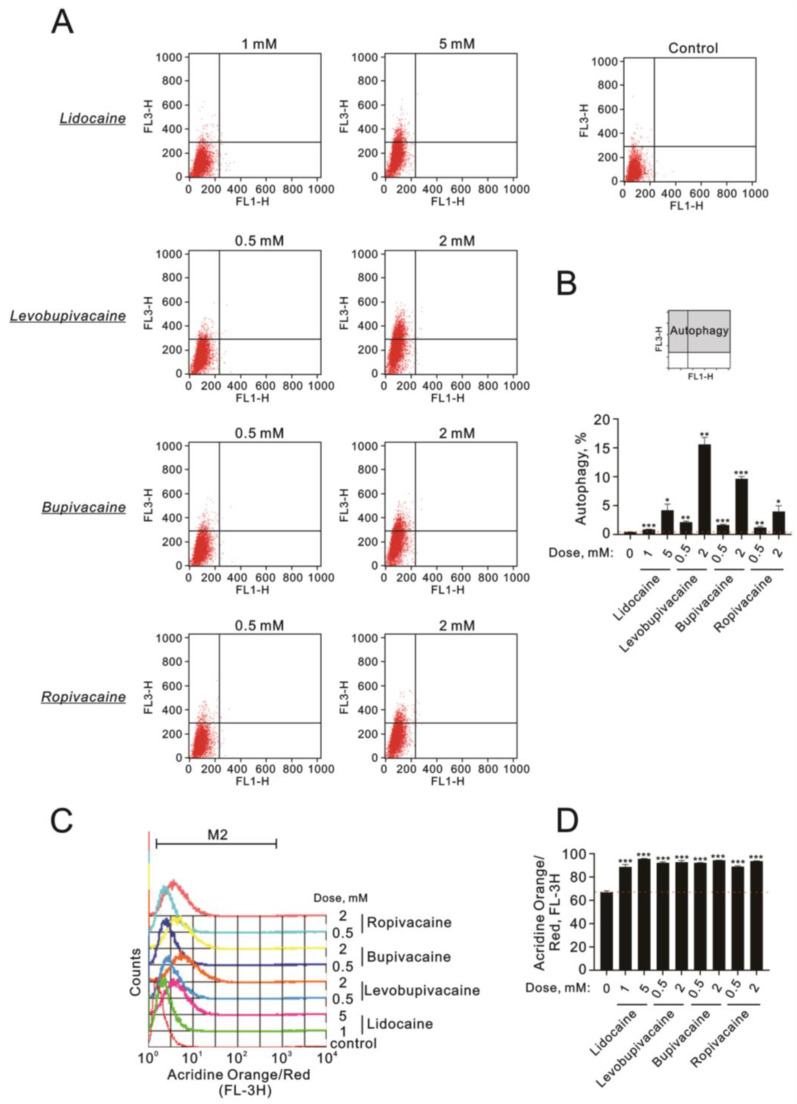
The effects of local anesthetics on autophagy in human TC28a2 chondrocyte cells. (**A**–**D**) TC28a2 cells were treated with the specified concentrations of lidocaine (1 and 5 mM), levobupivacaine (0.5 and 2 mM), bupivacaine (0.5 and 2 mM), and ropivacaine (0.5 and 2 mM) for 24 h. Acridine orange (1 µg/mL) staining was employed to identify autophagic cells through FACS analysis. (**A**,**B**) Acidic vesicular organelles were detected and quantified using acridine orange staining, with measurements obtained via flow cytometry. (**C**,**D**) The intensity of the red fluorescence (y-axis, FL3-H) was proportional to the acidity and volume of acidic vesicular organelles, including autophagic vacuoles. The values represent the percentages of cells containing a significant proportion of acidic vesicular organelles. The results presented in (**B**,**D**) are representative of three independent experiments. The dashed red line indicates the level for the vehicle control. The bars depict the means ± SDs. * *p* < 0.05; ** *p* < 0.01; *** *p* < 0.001 (Student’s *t*-tests).

Additionally, it is well-established that local anesthetics can trigger autophagy [26]. Our results illustrate that all tested anesthetics significantly promoted autophagy, with levobupivacaine (2 mM) and bupivacaine (2 mM) showing the strongest induction (Figure 6A). Collectively, these findings indicate that all examined local anesthetics effectively trigger autophagy in TC28a2 cells (Figure 6B).

## 3. Discussion

In orthopedic practice, local anesthetics can be administered through various routes—such as spinal, epidural, or intra-articular—to relieve postoperative pain or treat osteoarthritis. Recent studies have revealed that several commonly used local anesthetics exhibit toxicity to both human and animal chondrocytes, both in vitro and in vivo [24,25,27]. This study aimed to investigate the mechanisms underlying the cytotoxic effects of local anesthetics, focusing specifically on the impact of a single intra-articular injection on human chondrocyte cells. Our findings demonstrate that lidocaine, levobupivacaine, bupivacaine, and ropivacaine induced cell death, characterized by apoptosis and reduced cellular proliferation, which occurred through mechanisms involving oxidative stress, mitochondrial dysfunction, and autophagy pathways. The toxic effects of local anesthetics were found to be concentration-dependent, with lidocaine exhibiting the lowest cytotoxicity in TC28a2 cells. The detailed mechanisms by which local anesthetics suppress cellular proliferation require further investigation.

Our findings reveal that the cytotoxicity of the four local anesthetics in TC28a2 cells was ranked from highest to lowest as follows: bupivacaine, levobupivacaine, ropivacaine, and lidocaine. Notably, while lidocaine is known for its rapid onset and short duration of action, and bupivacaine for its slow onset and extended duration, our 24 h chondrocyte culture suggests that the duration of action may be the primary factor influencing the cytotoxic effects in human chondrocytes. In addition to lidocaine, one study demonstrated that ropivacaine may be a safer choice for intra-articular administration compared to levobupivacaine and bupivacaine [25]. The specific form of cell death induced by local anesthetics appears to be influenced by the duration of exposure, the concentration used, and the particular type of anesthetic. Research has shown that bupivacaine primarily leads to necrosis, while lidocaine predominantly triggers apoptosis [15]. Local anesthetics induced apoptosis, necrosis, and autophagy in a time- and concentration-dependent manner in vitro [27], compared with data showing that lidocaine, bupivacaine, and ropivacaine cause delayed mitochondrial dysfunction and apoptosis in cultured human chondrocytes [28]. Racemic bupivacaine exhibits enantioselectivity, with the R(+)-enantiomer demonstrating a more pronounced effect while levobupivacaine is the S(−)-enantiomer [29]. Levobupivacaine has similar potency to bupivacaine as a long-acting local anesthetic [30,31]. In clinical applications, bupivacaine is particularly favored for intra-articular analgesia due to its long-lasting effects. The introduction of levobupivacaine has the potential to reduce the risk of cardiovascular and CNS toxicity associated with bupivacaine. However, our 24 h treated chondrocyte cells showed that apoptosis was the primary cell death type, and only levobupivacaine had the ability to induce both necrotic and apoptotic cell deaths. Compared with bupivacaine, primarily in the induction of apoptosis and mitochondrial membrane depolarization, levobupivacaine had primary effects on the suppression of cellular proliferation and cytosolic ROS and the induction of autophagy. Our findings reveal that the topic of the benefits of bupivacaine and levobupivacaine in clinical applications is worth exploring.

Our current findings indicate that both bupivacaine and levobupivacaine demonstrate significant cytotoxic effects, particularly in relation to apoptosis, cellular proliferation, ROS production, mitochondrial membrane potential depolarization, and autophagy in human chondrocyte cells. The current development of protective agents may also reduce local anesthetic-induced cytotoxicity, including apoptosis inhibitors, inducers of cellular proliferation, modulators of mitochondrial membrane potential, mitochondrial ROS scavengers, and autophagy inhibitors [27,32,33]. In clinical practice, ultrasound-guided peripheral nerve blocks can help minimize the required dose of local anesthetics, thereby mitigating potential risks [34,35,36]. Therefore, these potential protective agents and novel technologies could broaden the clinical applications of local anesthetics. Further investigation is needed to determine whether adjunctive medications exacerbate or mitigate the chondrotoxic effects of local anesthetics.

## 4. Materials and Methods

### 4.1. Cell Culture and Reagents

The TC28a2 human chondrocyte cell line was obtained from Merck (Elkton, VA, USA). This cell line was created by transfecting primary cultures of costal cartilage from a 15 year-old female with a retroviral vector that expresses the simian virus SV40 large T antigen. TC28a2 cells were maintained in DMEM/F-12 medium (Corning, Corning, NY, USA) supplemented with 10% fetal bovine serum (FBS) and 1% penicillin-streptomycin (Thermo Fisher Scientific, Carlsbad, CA, USA), as previously described [37]. Bupivacaine, levobupivacaine, 2′,7-dichlorofluorescein diacetate (DCFH-DA), lidocaine, propidium iodide (PI), ropivacaine, and thiazolyl blue tetrazolium bromide (MTT) were purchased from Sigma-Aldrich (St. Louis, MO, USA).

### 4.2. Metabolic Activity Analysis

Cells were seeded in 24-well culture plates and incubated for 24 h. After this incubation period, fresh DMEM/F-12 medium containing bupivacaine, levobupivacaine, ropivacaine, and lidocaine was added to each well. Detailed procedural methods can be found in our previous publications [37]. The cells were then treated with bupivacaine, levobupivacaine, ropivacaine, and lidocaine for an additional 24 h. Following treatment, the cells were incubated with MTT solution at a concentration of 0.5 mg/mL for 1 h at 37 °C. After the incubation, DMSO was added to dissolve the formazan crystals formed during the reaction. Absorbance was measured at 570 nm and 650 nm using a multimode microplate reader (Varioskan™ LUX, Thermo Fisher Scientific), as previously described [38]. The metabolic activity was calculated based on the absorbance ratio between cells treated with the selected drugs and the untreated controls, which were assigned a value of 100.

### 4.3. Fluorescence-Activated Cell Sorting (FACS) for Flow Cytometry Analyses of Cell Cycle Profiles, Apoptosis, Cellular Proliferation, Cytosolic and Mitochondrial ROS, and Mitochondrial Membrane Potential

Cell cycle profiles were assessed by measuring cellular DNA content through PI staining. Following treatment with the drugs, the cells were trypsinized and washed with PBS. The cell pellet was then resuspended in 1 mL of PBS and fixed in 5 mL of 70% ice-cold ethanol before being stored at −30 °C overnight. The next day, the cells were washed twice with ice-cold PBS containing 1% FBS and centrifuged at 4 °C at 1000 rpm for 5 min. Subsequently, they were stained with a PI staining solution (5 μg/mL PI in PBS, 0.5% Triton X-100, and 0.5 μg/mL RNase A) for 30 min at 37 °C in the dark. Each sample was then analyzed using the FACSCalibur flow cytometer and Cell Quest Pro software (version 5.1, BD Biosciences, Milpitas, CA, USA), as previously described [39].

The early and late stages of apoptotic cells were assessed using a fluorescein PE–annexin V Apoptosis Detection Kit (BD Biosciences). Following the manufacturer’s protocol, cells were stained with PE–annexin V and 7-AAD to evaluate the effects of the specified concentrations of local anesthetics on necrosis (characterized by PE–annexin V-negative and 7-AAD-positive cells), early apoptosis (characterized by PE–annexin V-positive and 7-AAD-negative cells), and late apoptosis (characterized by PE–annexin V-positive and 7-AAD-positive cells), as previously described [38].

Cellular proliferation was evaluated using immunofluorescent staining with incorporated bromodeoxyuridine (BrdU) from the BD Pharmingen™ BrdU Flow Kit, following the manufacturer’s instructions. In brief, cells were seeded in 6-well culture plates and treated with the selected drugs for 24 h. After incubation, the cells were stained with BrdU, harvested, washed with PBS, and then fixed and permeabilized. Subsequently, they were stained with fluorescent antibodies specific to BrdU. The cells were resuspended in staining buffer, and fluorescence analysis for FITC-BrdU was conducted using the FACSCalibur flow cytometer and Cell Quest Pro software (version 5.1, BD Biosciences), as previously described [38].

The levels of cytosolic ROS and mitochondrial ROS were evaluated using DCFH-DA and MitoSOX Red staining, respectively. In brief, treated cells were washed twice with PBS and then incubated at 37 °C for 30 min in the dark with 10 μM DCFH-DA or 5 μM MitoSOX Red. Following this incubation, the cells were washed once more with PBS, and the fluorescence intensity of DCFH-DA or MitoSOX Red was analyzed using the FL-1 or FL-3 channel of the FACSCalibur flow cytometer and Cell Quest Pro software (version 5.1, BD Biosciences). The median fluorescence intensity of the vehicle control was used as the baseline for M1 gating, as previously described [37].

Mitochondrial membrane potential was assessed using the MitoScreen (JC-1) kit from BD Pharmingen. Following treatment with local anesthetics, both viable and dead cells were collected, and JC-1 solution was added to the samples for a 15 min incubation period. After incubation, the cells were thoroughly washed twice with a binding buffer to remove any excess dye. Each sample was then analyzed using the FACSCalibur flow cytometer in conjunction with Cell Quest Pro software (version 5.1, BD Biosciences), allowing for precise evaluation of mitochondrial membrane potential based on JC-1 fluorescence signals, as previously described [39].

### 4.4. Flow Cytometric Quantification of Acidic Vesicular Organelles

The acidic compartments of the cells were identified using acridine orange staining (Sigma-Aldrich, Cat. No. A8097) and measured via flow cytometry. As the protonated form of acridine orange accumulates within acidic vesicles, it serves as a marker for the later stages of the autophagy process. In brief, the cells were treated with the specified dosages of local anesthetics for 24 h and then stained with acridine orange (1 μg/mL) for 20 min at 37 °C before being trypsinized for harvesting. Following this, the cells were washed once with PBS and resuspended in 400 μL of PBS for flow cytometry analysis (FACSCalibur, BD Biosciences). The excitation wavelength was set to 488 nm, and fluorescence was detected at 510–530 nm (green fluorescence, FL1) and 650 nm (red fluorescence, FL3). Data analysis was performed using the Cell Quest Pro software (version 5.1, BD Biosciences). The percentage of autophagic cells was determined based on the number of cells located in the upper-left and upper-right quadrants, as previously described [37].

### 4.5. Statistical Analysis

The values are expressed as the means ± SDs of at least three independent experiments. All the comparisons between groups were conducted using Student’s *t*-tests with SPSS 20.0 for Windows (SPSS, Chicago, IL, USA). The statistical significance was set to *p* < 0.05.

## 5. Conclusions

Our findings demonstrate that lidocaine, levobupivacaine, bupivacaine, and ropivacaine induced cell death in human chondrocyte cells through mechanisms involving apoptosis, the suppression of cellular proliferation, oxidative stress, mitochondrial dysfunction, and autophagy pathways. The toxic effects of these local anesthetics were found to be concentration-dependent, with bupivacaine exhibiting the highest cytotoxicity, followed by levobupivacaine, ropivacaine, and lidocaine in TC28a cells. Our findings support current clinical studies and provide potential targets for the development of protecting agents to avoid the serious side effects of local anesthetics in orthopedic practices.

## 6. Limitations

The TC28a2 human chondrocyte cell line was created by transfecting primary cultures of costal cartilage with a retroviral vector expressing the SV40 large T antigen. In orthopedic practice, local anesthetics are administered via spinal, epidural, or intra-articular routes to manage postoperative pain and treat osteoarthritis. Consequently, the TC28a2 cells serve as a model for assessing chondrocytic toxicity induced by local anesthetics, rather than representing actual clinical target cells. Moreover, the concentration and duration of local anesthetic exposure in the cells may yield varying results. Therefore, the current conclusions should be approached with caution when making clinical decisions. 

## Data Availability

Data associated with the publication are available upon request from the corresponding author.
